# In Silico identification of M. TB proteins with diagnostic potential

**DOI:** 10.1186/1471-2172-14-S1-S9

**Published:** 2013-02-25

**Authors:** Romel Calero, Mayelin Mirabal, Jesús Bouza, María V Guzmán, Humberto Carrillo, Yamilé López, Mohd Nor Norazmi, Maria E Sarmiento, Armando Acosta

**Affiliations:** 1 Finlay Institute, Havana, Cuba; 2 Faculty of Sciences and Center for Complexity Sciences, National Autonomous University of Mexico, Mexico City, Mexico; 3 School of Health Sciences, Universiti Sains Malaysia, Malaysia; 4 Institute for Research in Molecular Medicine, Universiti Sains Malaysia, Malaysia

## Abstract

TB, caused by *Mycobacterium tuberculosis* (MTB), is one of the major global infectious diseases. For the pandemic control, early diagnosis with sensitive and specific methods is fundamental. With the advent of bioinformatics’ tools, the identification of several proteins involved in the pathogenesis of TB (TB) has been possible. In the present work, the MTB genome was explored to look for molecules with possible antigenic properties for their evaluation as part of new generation diagnostic kits based on the release of cytokines. Seven proteins from the MTB proteome and some of their combinations suited the computational test and the results suggested their potential use for the diagnosis of infection in the following population groups: Cuba, Mexico, Malaysia and sub-Saharan Africa. Our predictions were performed using public bioinformatics tools plus three computer programs, developed by our group, to facilitate information retrieval and processing.

## Introduction

MTB is the causative agent of TB. The only TB vaccine available, Bacille Calmette-Guérin (BCG) has been administered to more than one billion people and is routinely given to infants (not infected with HIV) worldwide. Although BCG provides a considerable degree of protection against pediatric TB, it does not protect people with HIV and it is unreliable against adult forms of pulmonary TB [1].

The association of TB with HIV/AIDS has dramatically increased the incidence of this disease. One-third of the world's population is infected with MTB and approximately 9 million people develop the active form of the disease every year with nearly 2 million deaths. It has been estimated that if no improvements in TB control are made, about 10 million people will die from TB by 2015 [2].

Early diagnosis is fundamental to prevent TB transmission and to minimize the risk of disease progression. The majority of patients are detected at an advanced stage of the disease, after having transmitted it to their closest contacts [3]. However, the development of new diagnostics poses great challenges to the scientific community, since our understanding of many of the underlying biological processes remains incomplete, and suitable biomarkers are yet to be identified. The identification of bacterial and/or host molecules that differentiate between people with active TB, those with latent infection and individuals not affected by TB are of priority to drive critical innovation, including the development of diagnostic tests with greater sensitivity and specificity [2].

New diagnostic methods include those based on the measurement of cytokine concentration such as interferon-gamma, released by the stimulation of blood lymphocytes [3]. The improvement of this type of diagnostic tests depends on the availability of antigens that provide greater sensitivity and ensure a specific response.

In the present work, using in silico methodologies, a group of MTB proteins were identified with the potential to induce the production of cytokines by the lymphocytes of MTB infected individuals without the production of cross reactive responses against BCG, *Mycobacterium bovis* (Mb) or environmental mycobacteria. The selected bacterial molecules were evaluated in silico to determine its biological function, sub-cellular location and its expression in vivo. Moreover, epitopes associated with the selected antigens were identified and their degree of presentation by different human populations was predicted.

## Material and methods

### Biological information sources

The whole genomic sequences of MTB H37Rv [4], Mb AF2122/97 [5] and the regions of differences found by Behr between MTB H37Rv, Mb and BCG [6] were used.

### Informatics and computational resources

Comparisons of sequences of MTB H37Rv against whole sequenced genomes of other MTB strains, Mb AF2122/97, *M. smegmatis* (Ms) and *M. avium* (Ma) were made with local sequence alignment tools: tfastax from FASTA 36.3.4 [7,8] and tblastn from BLAST 2.2.25+ [9].

Subcellular localization was predicted using Tbpred, SignalP and PSort servers [10-12]

HLAPred server was used to carry out the prediction of T cell epitopes corresponding to the selected proteins of MTB H37Rv, and the “Population Coverage Calculation” server [13] allowed the estimation of the theoretical population coverage of these epitopes in several geographic areas of interest.

The MTB antigens expressed in vivo in different species was determined by bibliographic search using google scholar, PubMed and PubMed Central.

Our bioinformatics approach was based on the use of already available bioinformatics resources combined with three auxiliary computational programs: NCBIReader, EBIFASTAProcessor and EpiFormat developed by our group (Calero R et al, unpublished results).

## Results and discussion

Behr and colleagues identified 16 regions of differences (RD1-RD16) between MTB H37Rv, Mb and BCG which encompasses a total of 129 open reading frames (ORF); we refer to them as “Behr’s regions”. Eleven of these regions are absent from Mb; the other five regions are present in Mb but absent from BCG.

We choose as starting point 100 ORFs that comprise twelve of the Behr´s regions. Four regions were discarded (RD2, RD8, RD14 and RD16) because they are present in some strains of BCG, in order to avoid cross reactivity with any BCG strain.

When comparing the genome sequences of Mb AF2122/97 with the 100 ORFs of MTB H37Rv, eleven ORFs had no significant alignments with Mb.

These 11 ORFs were compared with three sequenced strains of MTB (MTB F11, MTB KZN 1435 and MTB CDC1551). With the exception of one ORF (Rv3428c), all of them exhibited significant alignments and identities above 98% with each of the three strains of MTB; the ORF Rv3428c had only a single significant alignment with MTB F11. None of the 10 remaining ORFs gave significant alignments with the genome of Ms, but three ORFs (Rv2657c, Rv2348c and Rv1514c) gave significant alignment with Ma and therefore were discarded. The seven finally selected ORFs are shown in Table 1.

**Table 1:** Selected proteins

ORF	Description	Region	Subcellular location	*In vivo* expression		
				
				Human	Mice	Guinea Pig
Rv2658c	Prophage protein	RD13	Cytoplasmic			
Rv2655c	Prophage protein *phiRv2*	RD13	Cytoplasmic			
Rv2653c	Prophage protein *phiRv2*	RD13	Cytoplasmic			
Rv2645	Hypothetical protein	RD13	Cytoplasmic	X	X	
Rv1509	Hypothetical protein	RD6	Membrane			
Rv1507c	Hypothetical protein	RD6	Cytoplasmic			
Rv1508c	Hypothetical protein	RD6	Membrane	X	X	X

From these seven ORFs a collection of T cell epitopes was identified, establishing the position in which they are located in the amino acid sequence of the protein, the group of alleles that has affinity for each of them, and the corresponding degree of affinity (prediction score). It was also possible to identify promiscuous epitopes and their corresponding alleles. This knowledge, together with the full sequence of each epitope, served to theoretically estimate the potential population coverage of the selected antigens. In turn, the knowledge of the population coverage served to calculate an a priori estimate of the potentiality of the antigens (or their combinations), to be used in different geographical regions. Coverage of combination of two or three of the selected protein sequence was also predicted. Focusing on the Cuban population, the information contained in previous works [14-17] was useful to create an updated version of the allele database contained in the Population Coverage Calculation Server.

**Figure 1:**
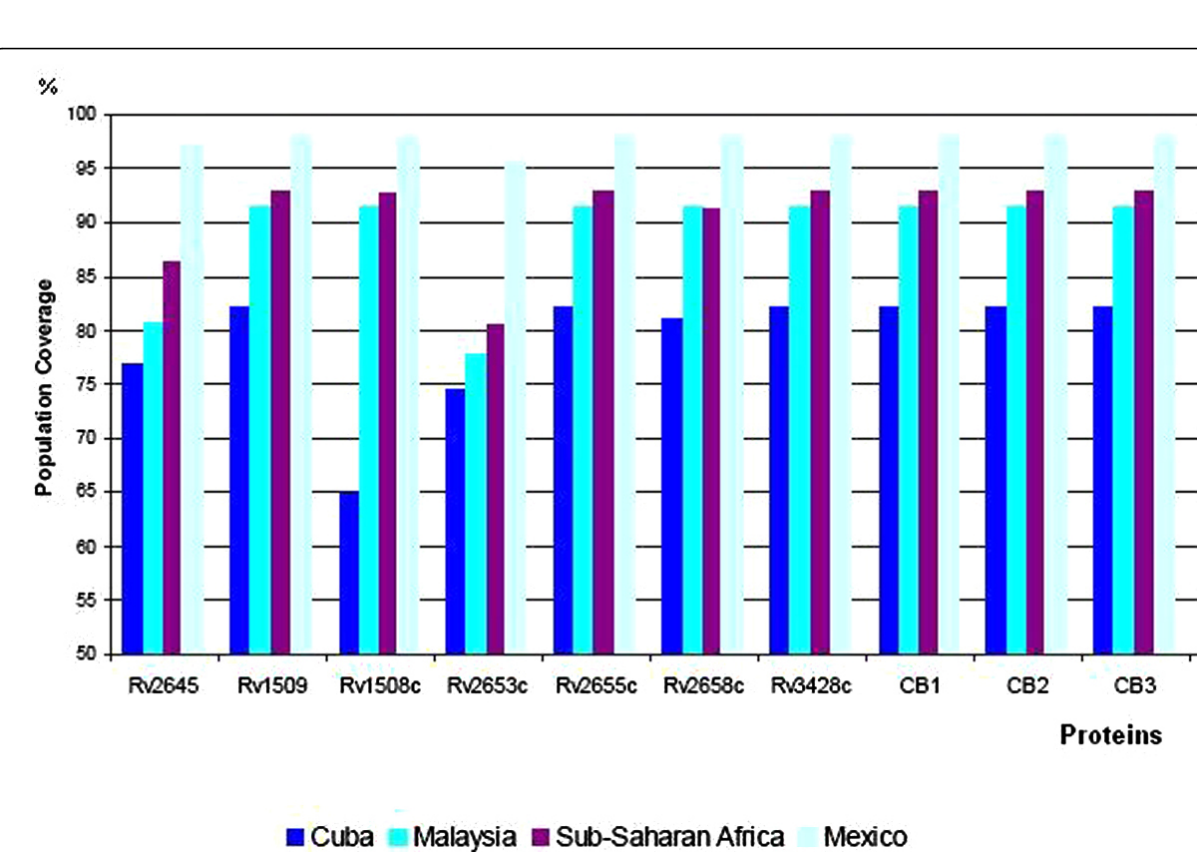
Population coverage (HLA Class I & Class II) of seven selected proteins (listed on Table 1) and three of its combinations (CB1: Rv1509+Rv1508c; CB2: Rv2645+Rv1509; CB3: Rv2658c+Rv1508c), for the tested populations.

The theoretical predictions of the coverage (HLA Class I & Class II) are summarized in Figure 1. Four proteins and three of their combinations offer coverage of more than 80% for all the tested populations.

The bioinformatics strategy proposed and used here has rendered positive results: several molecules that have promising antigenic properties to be used in the development of diagnostic tests for TB, have been identified. Our selection procedure theoretically guarantees that these antigens have the potential to offer an adequate degree of specificity to recognize the MTB infection, minimizing the likelihood of false positive, in conditions that are typical in the geographical areas of high incidence of TB: vaccination with BCG, Mb infection or infections with environmental mycobacteria [18,19]. These predictive results should be confirmed in future human studies carried out in different populations.

## Competing interests

The authors declare that they have no competing financial interests.

## Authors' contributions

All authors have read and approved the final manuscript. RC developed softwares used in the bioinformatics studies, performed the bioinformatics studies and participated in data analysis and in writing of the manuscript, MM, JB participated in the bioinformatics studies and data analysis, MVG, HC participated in the bioinformatics studies, data analysis and in writing of the manuscript, YL writing of the manuscript, MNN, MES, AA conceived the study, participated in the bioinformatics studies, in data analysis and in writing of the manuscript.

## Declarations

This article has been published as part of *BMC Immunology* Volume 14 Supplement 1, 2013: Proceedings from Delivery Systems and Current strategies to drug design. The full contents of the supplement are available online at http://www.biomedcentral.com/bmcimmunol/supplements/14/S1
